# Supplementary material for: The Influencing Factors of Aggregation Behavior of Tree-of-Heaven Trunk Weevil, Eucryptorrhynchus brandti (Harold) (Coleoptera: Curculionidae)
Source: Insects. 2023 Mar 3;14(3):253. doi: 10.3390/insects14030253 (PMC10054803; doi:10.3390/insects14030253)
Supplement: Supplementary file 1 [file insects-14-00253-s001.zip › insects-2228803-supplementary.pdf]

Supplementary Materials:

Formulas S1–S3

Rr = $\frac{a+b}{c}$ ×100% (S1)

SRr = $\frac{a}{a+b} \times 100\%$ (S2)

SC $= \frac{a-b}{a+b} \times 100\%$ (S3)

Formulas S1–S3. Calculated Rr, SRr, SC, including that a: number of adults in the odor source tube; b: number of adults in the air tube; c: total number of test adults.
